# Case Report: Tubridge flow diverter for a ruptured fusiform aneurysm of the M1 segments of the middle cerebral artery

**DOI:** 10.3389/fsurg.2022.941355

**Published:** 2022-10-25

**Authors:** Sen Wei, Jingjing Wang, Xinbin Guo, Sheng Guan

**Affiliations:** Department of Neurointervention, The First Affiliated Hospital of Zhengzhou University at Zhengzhou, Zhengzhou, China

**Keywords:** aneurysm, reptured, tubridge flow diverter, fusiform, middle cerebral artery

## Abstract

We report a case of the middle cerebral artery (MCA) M1 segment ruptured fusiform aneurysm that was successfully treated using a domestic Tubridge flow diverter (TFD). A 40-year-old man was admitted to the hospital because of a headache and was diagnosed with subarachnoid hemorrhage. Cerebral angiography revealed a ruptured fusiform aneurysm in the M1 segment of the right MCA. TFD, combined with coil embolization, was used for perioperative treatment. No obvious complications were observed. Follow-up digital subtraction angiography 2 and 12 months after the surgery showed that the aneurysm was occluded, and the patient recovered well. This is the first known case of this treatment with this type of stent in such an aneurysm and demonstrates that TFD can be used to treat ruptured fusiform aneurysms in the M1 segment of the MCA.

## Introduction

Intracranial fusiform aneurysms are difficult to treat and carry a higher risk of rebleeding and morbidity than saccular aneurysms (1). The emergence of a flow diverter (FD) provides a new option for the endovascular treatment of fusiform aneurysms. Several studies have reported that FD can effectively treat middle cerebral artery (MCA) aneurysms, including pipeline flow diverters (PFD) and domestic Tubridge flow diverters (TFD) (2–5). However, there are a few studies on the application of FD to ruptured fusiform aneurysms of the MCA. A previous case report demonstrated that the use of PFD in the treatment of a ruptured fusiform aneurysm in the MCA was successful, and the short-term follow-up results were good (6), while TFD in the treatment of MCA ruptured cases of fusiform aneurysms have not been reported in the literature. We report a case of a patient with a ruptured fusiform aneurysm in the M1 segment of the MCA that was successfully treated using TFD combined with coil embolization in October 2020.

## Case presentation

**Figure 1:**
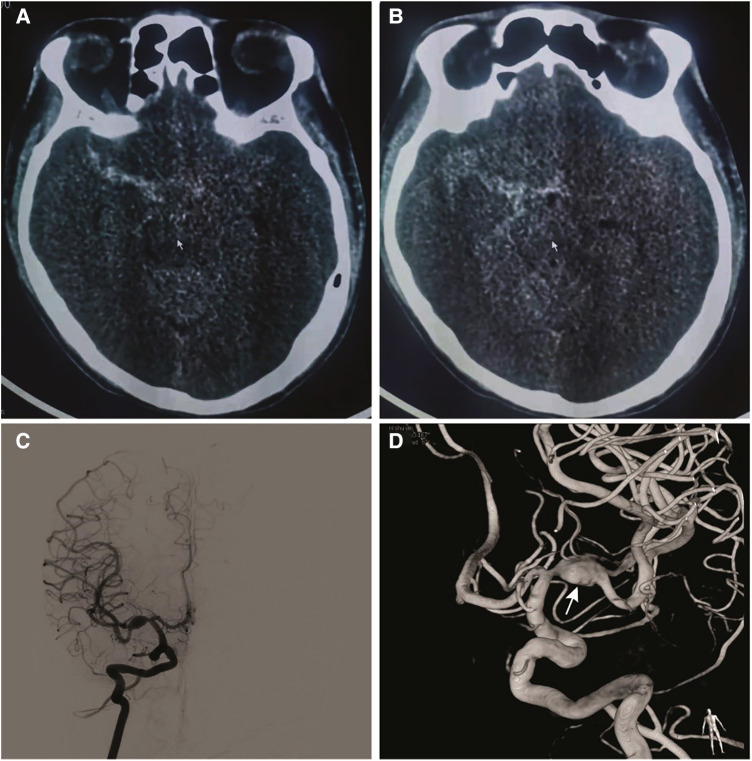
(**A**) and (**B**) computed tomography of the head demonstrating subarachnoid hemorrhage. (**C**) Anterior-posterior the right internal carotid artery and (**D**) 3D rotational angiogram demonstrating a fusiform aneurysm of the M1 segment of the right MCA measuring 10.11 × 6.47 mm. Multiple ascuses (arrow) were visible in the inferior wall of aneurysm.

A 40-year-old man presented with a headache, followed by a low mental state. An emergency computed tomography (CT) scan of the head revealed a subarachnoid hemorrhage (Figures 1A,B). The digital subtraction angiography (DSA) and preoperative third-dimensional rotational angiogram (Figures 1C,D) were performed to demonstrate a fusiform aneurysm measuring 10.11 × 6.47 mm in the right M1 segment of the MCA. There were multiple subsacs in the inferior wall, and a ruptured aneurysm was considered. Owing to fusiform aneurysms with high rebleeding and morbidity rates, after a discussion with his family regarding the treatment effect and prognosis, the decision was made to treat the aneurysm using TFD combined with coils. In addition, a neck pressure test was performed on the preoperative angiography of left internal carotid artery, which showed the opening of the anterior communicating artery.

## Treatment

**Figure 2:**
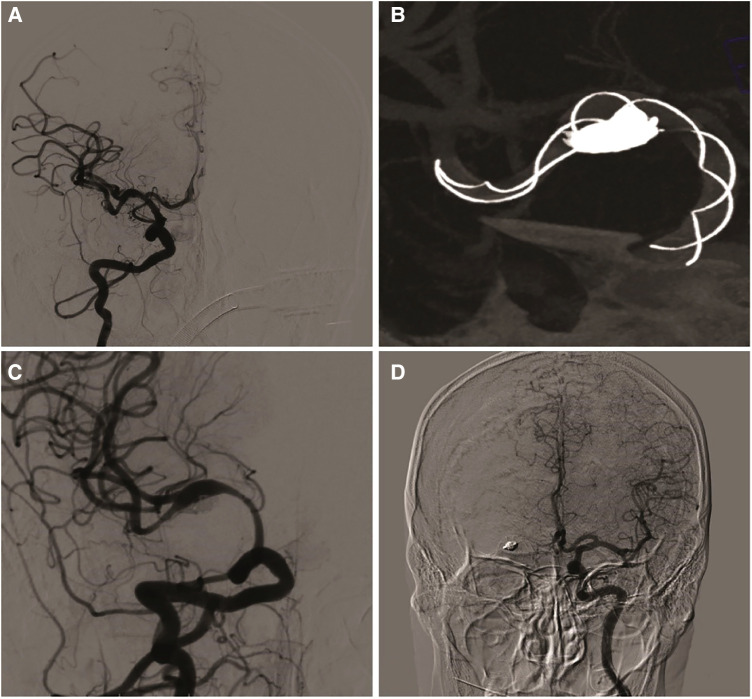
(**A**) patient was treated by embolization with TFD. (**B**) The stent is completely released and filled with coils through microcatheter. (**C**) two months later, angiographic follow-up showed partial occlusion of the aneurysm and moderate in-stent stenosis of the ipsilateral middle cerebral artery. (**D**) The anterior communicating artery was open and the ipsilateral anterior cerebral artery was well developed.

The interventional procedure was performed under general anesthesia and systemic heparinization. A 7F long sheath (90 cm, Cook Company, USA) was used as a supporting catheter, which was placed at the C1 end of the right internal carotid artery. With the cooperation of a 5F Navien catheter (115 cm, Medtronic, USA) and 0.014 micro-guide wire (200 cm, Stryker, USA), a T-track stent catheter (MicroPort, Shanghai) was introduced in the ipsilateral MCA M3. A Tubridge stent (4.0 × 45 mm, MicroPort, China) was introduced along the stent catheter, and an SL-10 (Excelsior, Stryker, USA) microcatheter tip was attached to the microguide wire. After shaping, it was introduced into the aneurysmal sac. At this time, the stent was released to the beginning of M1, and AXIUM coils (7 mm × 30 cm, 4 mm × 12 cm, 3 mm × 8 cm, and 2 mm × 8 cm; Medtronic, USA) were successively filled along the embolization catheter. The coil and stent were repeatedly adjusted to place the coil on the anterior and inferior walls of the MCA. When the ruptured aneurysm sac was densely filled, the stent was completely released. Angiography and stent CT showed that the stent was well opened, and a dense tamponade was performed on the coils on the anterior and inferior walls. (Figures 2A,B). After the aneurysmal sac was densely packed, 10 ml of tirofiban injection was administered intravenously and then pumped in a 6 ml L/h micropump. After 24 h of tirofiban administration, 300 mg of aspirin and 300 mg of oral clopidogrel were administered, after which tirofiban was still administered for 4 h. The infusion of tirofiban was discontinued, and antiplatelet therapy was administered at 75 mg of clopidogrel and 100 mg of aspirin daily. The patient's vital signs were stable during the operation, and he did not complain of discomfort after waking. Three days after the operation, no obvious abnormality was found on re-examination of the head MRI, and the patient was discharged 7 days after the operation. The modified Rankin Scale (mRS) score at discharge was 0. After discharge, the patient was informed of regular dual anti-platelet aggregation therapy (75 mg clopidogrel + 100 mg aspirin orally once a day) within 6 months. Two months after the operation, the patient had no obvious discomfort, and the mRS score was 0. Re-examination *via* DSA showed aneurysm occlusion, moderate in-stent stenosis at the distal and proximal ends of the stent, and occlusion of the A1 segment of the right anterior cerebral artery and opening of the anterior communicating artery (Figures 2C,D).

**Figure 3:**
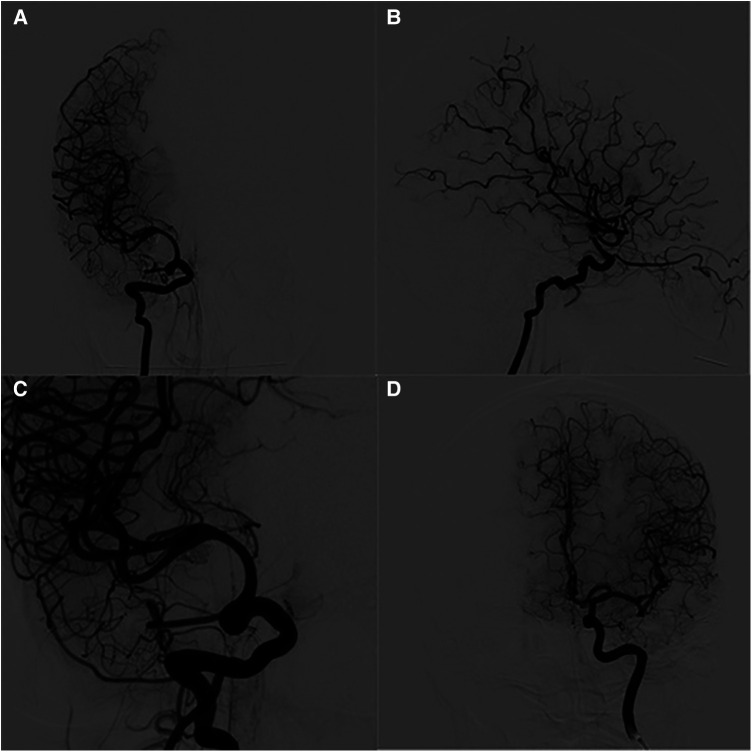
DSA images findings 12-months after operation. (**A**) anteroposterior angiography of the right internal carotid artery. (**B**) Lateral angiography of the right internal carotid artery. (**C**) The working position angiography showed that the stenosis at the distal and proximal ends of the stent disappeared. (**D**) The anteroposterior angiography of the left internal carotid artery showed that the anterior communicating artery was open and the ipsilateral anterior cerebral artery was well developed.

The patient has asymptomatic moderate in-stent stenosis. He was recommended to continue oral aspirin and clopidogrel for anti-platelet aggregation therapy. At 12 months after operation, the patient was followed up in our hospital. The patient had no obvious discomfort and the mRS score was 0. DSA showed that, anteroposterior (Figure 3A) and lateral (Figure 3B) angiography of right internal carotid artery demonstrated the right middle artery was smooth, but right anterior cerebral artery was not visualized. The working position angiography showed that the blood flow in the stent was smooth, and the stenosis at the distal and proximal ends of the stent disappeared. (Figure 3C) The anteroposterior angiography of the left internal carotid artery (Figure 3D) showed an open anterior communication with no obvious loss of staining in the area supplied by the right anterior cerebral artery. The patient was instructed to discontinue clopidogrel but remain to take 100 mg of aspirin, and recheck angiography after 2 years.

## Discussion

Ruptured fusiform aneurysms in the M1 segment of the MCA are relatively rare in clinical practice, and there is no consensus regarding the standard treatment regimen. Surgical craniotomy requires an isolated bypass, which is more traumatic. Considering that the lenticular artery originates from the M1 segment, the patient is likely to be disabled after surgery. In this case, after consultation with a neurosurgeon, surgical isolated bypass surgery was not recommended. Conventional stent-assisted embolization can embolize aneurysm sacs densely, but because of the low metal coverage of conventional stents, it is difficult to form a thrombus between the edge of the stent and the wall of the MCA lesion in a short period of time. Coupled with the application of antithrombotic drugs, there is a higher risk of rebleeding in the short term. Compared with conventional stent-assisted embolization, blood flow diverting devices may have higher safety, and there have been case reports of FD combined with coil embolization in the treatment of MCA ruptured fusiform aneurysms. The safety and short-term follow-up effects of the procedure are good (6). Based on the patient's condition and current treatment status, the patient was treated with FD combined with coil embolization.

The study of a flow-diverting device combined with coil embolization in the treatment of ruptured fusiform aneurysms of the MCA is limited to case reports. Moreover, PFD is used, and the postoperative follow-up time is short (6). To date, no reports on the use of TFD in the treatment of ruptured fusiform aneurysms in the MCA have been documented. To our knowledge, this is the first report of a patient with an MCA ruptured fusiform aneurysm treated using TFD combined with coil embolization. The patient recovered well after 1 year of follow-up, and the aneurysm had no obvious recurrence.

TFD is a new type of FD that was independently developed in China, and its safety and efficacy have been widely recognized (7). The patient had a ruptured fusiform aneurysm of the MCA. The coil is not only used to fill the ruptured anterior inferior wall but also as an effective support for TFD. Through TFD placement, the blood flow velocity and wall shear stress in the aneurysm are significantly reduced, which accelerated thrombosis between the TFD and the lesion wall, and achieves aneurysm occlusion and parent artery remodeling in the short term. Short-term follow-up also confirmed aneurysm occlusion, and long-term follow-up showed that the M1 segment of the MCA was well remodeled (8). Although short-term follow-up revealed moderate in-stent stenosis, the patient was asymptomatic. Previous literature has reported that, after FD treatment, most patients can tolerate reduced blood flow due to arterial occlusion or stenosis without significant associated symptoms or imaging findings (9). Benninghaus et al. reported that in-stent stenosis is a common phenomenon in the short-term follow-up after FD treatment, and the mechanism is unclear; however, most patients are asymptomatic, and the stenosis will improve with prolonged follow-up (10). In this case, moderate in-stent stenosis was found during the 2-month follow-up period, and regular dual anti-platelet aggregation therapy was continued. The stenosis disappeared during the 12-month follow-up, which is consistent with previous reports.

This article reports a successful case of the use of TFD, combined with coil embolization, in the treatment of a ruptured fusiform aneurysm in the M1 segment of the MCA, with good short-term and long-term follow-up. Future multicenter prospective cohort studies are needed to confirm this conclusion.

## Data Availability

The original contributions presented in the study are included in the article/Supplementary Material, further inquiries can be directed to the corresponding author/s.
